# SHEPHERD UNIVERSITY’S ROADMAP TO AGE-FRIENDLY UNIVERSITY DESIGNATION

**DOI:** 10.1093/geroni/igae098.1758

**Published:** 2024-12-31

**Authors:** Heidi Dobish

**Affiliations:** Shepherd University, Shepherdstown, West Virginia, United States

## Abstract

Our AFU dedicated team first met in August, 2022 to discuss the process for becoming an Age-Friendly University. The AFU team consisted of a psychology department faculty member, the director of the university’s Lifelong Learning Program, and a local community member. Our first step was to map our university’s core values and current age-friendly inclusive activities onto the 10 Principles of an Age-Friendly University. This exercise provided evidence that many ongoing, university-engaging events highlighted four of the Age-Friendly principles. In October, 2022, the team met with our provost and proposed why Shepherd University should endorse the Age-Friendly initiative. Our provost agreed and signed our application letter in November, 2022. With the Age-Friendly University Global Network restructuring, it was a rather long wait for their response but we received our acceptance letter in August, 2023. We sent out press releases and were interviewed by a local columnist. Our first step as an AFU member was to create a six-person AFU Advisory Board with representation from Shepherd faculty, staff, and community members. We meet monthly and are currently working on a mission statement, by-laws, and S.W.O.T. analysis. We are also conducting information sessions for the university community to inform them about AFU and receive input on potential age-friendly activities. The board is keeping a log of activities undertaken as required by the AFU Global Network.

